# Promoting Best Practices for Medical Science Liaisons Position Statement from the APPA, IFAPP, MAPS and MSLS

**DOI:** 10.1007/s43441-021-00310-y

**Published:** 2021-07-08

**Authors:** Paul Theron, Matthew Britland, Donna Holder, Yasushi Ikeda, Ralph F. Rewers, Ajay Tiku

**Affiliations:** 1 Head Medical Excellence APAC, Merck Healthcare Pty Ltd, 10 Mahratta Ave, Wahroonga, NSW 2076 Australia; 2 Medical Director, Amgen, Macquarie Park, Australia; 3 Senior Director, Global MSL Excellence Oncology, Daiichi Sankyo Inc, Basking Ridge, USA; 4 Vice President, Medical Science Liaison, Astellas Pharma Inc, Tokyo, Japan; 5 National MSL Director, US Medical Affairs, AbbVie, Chicago, USA; 6 Regional Medical Head, Asia Pacific, Middle East and Africa, Novartis, Singapore, Singapore

**Keywords:** Medical Science Liaison, Role, Standards, Communications, Interaction, Industry

## Abstract

This position paper is intended to provide recommendations that will help lay the foundation for best practices for medical science liaisons (MSLs) and their activities. Its objective is to outline the roles and responsibilities expected of an MSL and provide clarity on the juxtaposition of MSLs and Sales representatives (SRs) when it comes to scientific exchange versus promotional messaging. It is of utmost importance that industry integrity and ethical standards are assured during external stakeholder engagement as well as medical and scientific communications. This guidance, delivered through the lens of APPA, IFAPP, MAPS and the MSLS executive committees, has been prepared primarily as a supportive resource to assist the Medical Affairs teams in the industry to develop their own set of standard operating procedures (SOPs), codes of conduct and policies within the framework of relevant industry regulations. We acknowledge that whilst there are guidelines already available that provide excellent directive to the MSL function, this paper is a review and distillation of these existing recommendations combined with the perspectives of four peak professional bodies to offer a practically focused resource to help MSLs interact, collaborate and exchange scientific information appropriately with external experts when out in the field.

## Introduction

The medical science liaison (MSL) is a key member of the Medical Affairs team, a department within the pharmaceutical organisation that is involved in the communication of accurate and unbiased scientific and medical information to healthcare professionals [1, 2]. Such communications typically involve safety information or updates, published papers, independent medical education, as well as responses to requests for off-label information [2] .However, more recent times has seen an increased need for MSLs but an accompanying reduction in sales and marketing personnel [3]. Consequently, there has been an emergence of hybrid roles that combine medical affairs and sales roles and consequently ‘muddy the waters’ when it comes to separating scientific and commercial activities. A lack of a global perspective on the role of the MSL [4–6], combined with limited published literature by experts in Medical Affairs [1, 7], as well as white papers developed by health consulting firms with a commercial interest [8–12], mean that there is no formal framework defining the roles and responsibilities of an MSL.

Recognising this gap, the executive committees of four peak body organisations representing Medical Affairs’ members: the Australian Pharmaceutical Medical and Scientific Professionals Association (APPA), the International Federation of Associations of Pharmaceutical Physicians and Pharmaceutical Medicine (IFAPP), the Medical Affairs Professional Society (MAPS) and the Medical Science Liaison Society (MSLS), provide an unbiased, commercial-free narrative review and recommendations on key practices of an MSL. It is the hope of the authors that this paper will help to ensure the future integrity of the key Medical Affairs function.

## Methodology

A literature review was conducted using PubMed where searches were performed using search string variations of the following keywords: (medical affairs*, medical science liaisons*, role*, responsibilities*, activities*). Google Scholar citation searches and manual searches of reference lists were also conducted to identify relevant publications, as well as general Google searches using the same search strings above. Searches were performed between May 2020 and July 2020 with only those retrieved records published in the last 5 years. The list of sources of information used by the authors is provided in the reference section.

Recommendations and key principles were developed by the author representatives of the peak bodies. APPA is the representative association for Medical Affairs in Australia and is dedicated to promoting excellence in pharmaceutical medicine through professional development, networking and advocacy (http://appa.net.au); IFAPP’s primary objective is to bring together physicians and scientists from the pharmaceutical industry and contract research organisations with colleagues working in research institutes, academia, medicines regulatory agencies and patient organisations, in order to stimulate the advancement of knowledge in Pharmaceutical Medicine globally (https://ifapp.org/);

MAPS is the premier non-profit global Medical Affairs organisation FOR Medical Affairs professionals BY Medical Affairs professionals across all different levels of experience/specialty to engage, empower and educate. Together with over 3,000 Medical Affairs members from over 200 companies, MAPS is transforming the Medical Affairs profession to increase its value to patients, HCPs and other decision makers (https://medicalaffairs.org); and MSLS is the only non-profit organisation focused exclusively on the global MSL profession. The MSL Society serves as a voice for the global MSL profession and represents members in 83 countries. The organisation is dedicated to advancing the profession by helping MSLs and MSL leaders become more effective in their careers through focused training programs, global research and best practice sharing (www.themsls.org). Given the wide reach of each of these organisations, the authors had access to a large number of member insights, activities and perspectives.

## What is the Role of the MSL?

The MSL has a key role in developing and delivering scientific communications to health professionals and other stakeholders that is factually accurate and compliant with industry standards.

The MSL role has existed for more than 50 years and continues to evolve in line with changing disease, treatment landscapes and healthcare trends. The ability to translate scientific research to clinical practice (i.e. from the bench to the bedside) remains a fundamental skill for all MSLs and is key to understanding, interpreting and discussing therapeutic advancements. MSLs represent the scientific face and force of the pharma industry, connecting companies with the medical community that include a range of stakeholders, such as key opinion leaders, clinical investigators and healthcare decision makers. Through the exchange of highly credible, unbiased, scientific and clinical information, MSLs can build and foster important scientific credibility with these external experts.

Another important and large part of the value proposition of the MSL is being able to bring relevant, timely and actionable insights to the company. Through such communication and collaboration, the ultimate objective is to help the right patients gain access to the right medicines.

To this point, MSLs are well positioned to inform on the safe and appropriate use of therapeutics. Over time, MSLs have needed to become increasingly versatile given the emergence of new and broader groups of stakeholders, a shift to a more patient-centric model of care where the focus is less about the drug and more about the patient journey and technological advances in diagnostics, devices and treatments. As the healthcare landscape continues to evolve, MSLs need to keep abreast of advancements and understand how they may impact clinical practice and patient outcomes.

It is important to acknowledge that variations exist in the MSL role including the name of the role. This is illustrated within the MSL Society guidelines, which outlines more than 30 titles (Table 1). Therefore, it is necessary for the company to clearly define and communicate the MSL’s roles and responsibilities.

**Table 1:** Alternative titles for an MSL (note this is not an exclusive list)

Clinical Liaison	Medical Development Advisor	Precision Medicine Liaison
Clinical Science Consultant	Medical Liaison	Regional Medical Advisor
Clinical Science Liaison	Medical Liaison Manager	Regional Medical Director
Clinical Science Specialist	Medical Manager	Regional Medical Liaison
Clinical Specialist	Medical Outcomes Liaison	Regional Medical Manager
Clinical Trial Educator	Medical Relationship Science	Regional Medical Scientific
Clinical Trial Liaison	Medical Science Consultant	Regional Scientific Manager
Field Medical Director	Medical Science Manager	Remote Medical Liaison
Global Medical Advisor	Medical Scientific Director	Scientific Affairs Manager
Market Access Liaison	Medical Scientist	
Medical Advisor	Molecular Science Liaison	

Reproduced with the permission from the MSLS

## What Are the Responsibilities of MSLs?

MSLs are responsible for scientific exchange of data, supporting evidence generation and gathering actionable insights from the field.

### Scientific Exchange

MSLs play a role in the exchange of unbiased scientific information when delivering presentations and education relating to the disease, unmet needs, treatment landscape, therapy areas and clinical evidence. They also engage and liaise with experts on the generation of scientific data. MSLs can proactively seek an introductory meeting with an external expert for the purpose of determining unmet medical needs and assessing the opinion leader’s criteria for and interest in scientific engagement. Subsequent or follow-up meetings, however, also need a purpose and a clear objective, requiring the MSL to clarify the scientific need for the exchange with the HCP.

### Evidence Generation

Evidence generation activities include company-sponsored studies, investigator-initiated trials, real-world evidence studies and clinical audits. To facilitate these, MSLs require appropriate training in good clinical practice and an understanding of the appropriate clinical trial standard operating procedures (SOPs) and protocols. Of note, health economics and outcomes research (HEOR) studies represent a developing intersection between medical affairs, market access and commercial teams.

### Gathering Insights

MSLs are well positioned to gather insights from the field that can be used to inform internal clinical development, marketing and market access in developing their strategies. These insights may be based on expert opinion, observations of barriers in the patient journey or questions that emerge in scientific exchange. Insights may also be gained from advisory board meetings.

If during interactions with external experts, concerns about the safety of the product are raised, or adverse events in patients treated with the product are mentioned, irrespective of whether the event was considered to be drug-related or not, the MSL should submit the information as per the company’s pharmacovigilance policy on adverse event reporting.

Key Principles

- MSLs alongside the wider Medical Affairs team should identify experts according to their medical expertise and academic reputation within the field of specialty; quality of publications; positions in peak bodies/societies/association; relevance of their level of clinical expertise, participation in treatment guideline bodies, as well as participation in research including clinical trials
- Selection of experts should not be based on their potential to prescribe or number of patients they see
- MSLs should develop and maintain strong and constructive relationships, at the same time respecting the independence of the key external experts
- MSLs may share insights from the field with medical and appropriate members of marketing teams to assist with the development of research strategies, medical communications, medical plans, brand plans, launch plans and materials
- MSLs play a key role throughout the lifecycle of a product: clinical development, pre-launch, launch and post-launch
- Scientific exchange should never contain false or misleading information, or omit or select information which by default could lead to misleading the stakeholder
- MSLs should not provide patient specific treatment advice or discuss specific patients
- MSLs should report adverse events raised during external stakeholder interaction within 24 h

## What Are the Core Skills Required by MSLs?

Core requirements of an MSL are to hold an advanced degree, demonstrate scientific and technical expertise, be excellent communicators and have strong interpersonal skills.

Given the primary role in building and fostering peer-to-peer credibility with key external stakeholders, a strong scientific and medical grounding, as well as excellent communication and interpersonal skills are essential. MSLs also need to have sound business acumen and leadership qualities. Typical academic qualifications for MSLs include medical, pharmacy and scientific PhD degrees.

Whilst these criteria remain the foundation for MSLs, additional skillsets are also important. These include value skills, reflected in the ability to engage external partnerships, for example, integrated delivery networks and payors, understand outcome-based agreements, as well as develop medical strategies that support drug access objectives. Being able to gather, assimilate and interpret information appropriately and feeding that through to the relevant internal teams is also a key competency of an MSL, as is having excellent data analytical skills. Digital competency is becoming increasingly important to allow for easier access to difficult to reach stakeholders and provides the MSL a means to engage with experts virtually if their area of coverage is wide.

Key Principles

- MSLs have a responsibility to support non-promotional activities in the field that encourage the safe and appropriate use of medicines
- Companies should upskill the MSLs with the necessary knowledge about the therapeutic area, the disease and its management
- The MSL is a Medical Affairs professional that acts as an important bridge between the company and the healthcare ecosystem by identifying clinical unmet needs, educating on the mode of action of new compounds as well as their efficacy and safety data whilst helping to identify appropriate patient profiles that might benefit from the new medicine. These discussions with key stakeholders should be conducted in a non-promotional, highly scientific and unbiased manner
- Combining professional qualifications with appropriate training will equip MSLs to convey medical and scientific principles accurately without bias and with transparency
- MSLs should have a strong understanding of the relevant industry codes including any country-specific and local policies relating to interactions with healthcare providers
- Companies should ensure the availability of internal training programs covering therapeutic and pipeline portfolios, healthcare system processes, reimbursement models, medical compliance and core skills (e.g. medical writing; critiquing scientific publications) to allow the MSLs to conduct their role effectively

## What is Meant by Non-promotional Activities and Interactions?

Non-promotional activities and interactions are those that have no commercial- or sale-based objectives.

A key differentiator between MSLs and SRs, both customer-facing roles, is that MSLs are only permitted to interact with external experts in a non-promotional context. This means that MSL activities should not be driven by prescription or sales targets, which represent benchmark metrics for SRs, but rather by indicators that demonstrate the need for scientific engagement as well as dissemination of data. At times, these two functions have become blurred. MSLs should not be perceived as product advocates but rather as scientific experts in their therapeutic field. Indeed, MSLs are a valuable internal resource for scientific training of sales and marketing teams, but appropriate steps need to be undertaken to ensure appropriate separation between commercial and Medical Affairs departments so as to avoid influence or the appearance of influence across the groups.

Key Principles

- MSLs need to maintain independence from sales and promotional-based activities
- MSLs are well positioned to gather insights from the field that can be used to help internal stakeholders to shape their strategies for improving patient outcomes
- MSLs and SRs typically only see healthcare professionals together for introductory visits. If during the meeting, conversations progress in a more commercial way, the MSL normally leaves the meeting. Conversely, if the conversation involves off-label discussions, it is appropriate for the sales colleague to leave the meeting
- Companies need to set clear guidance on the boundaries of commercial team participation in meetings with external experts, as well as their purpose at such meetings
- MSLs are permitted to share the list and schedule of opinion leader/external expert visits with commercial colleagues
- MSLs should not have sales targets as key performance indicators
- It is very strongly recommended that the MSL function reports into Medical Affairs to maintain their autonomy and demonstrate that theirs is primarily a non-promotional role and hence they may engage in appropriate, even off-label, scientific exchange
- MSLs should not engage in promotional messaging or participate in promotional discussions

## What Are the Common Compliance Concerns and How to Avoid Them?

### Unsolicited vs Solicited Requests and Off-Label Discussions

Overall, MSLs largely interact with medical and scientific experts, and HCPs in a planned manner within the framework of an ‘Expert Engagement Plan’. Whilst HCPs can prescribe drugs outside their intended use, it is illegal for any associate of the pharma industry to promote products for off-label use.

Examples of these proactive engagements include changes to the prescribing or product label; safety signals or concerns related to the therapeutic product; introductory meetings and insight gathering, as indicated earlier, in the form of advisory boards, working group meetings or roundtable discussions, as well as for non-product scientific education activities. Proactive engagements should never be for the purpose of providing off-label information.

MSLs may also respond to HCPs reactively. They respond to bona fide unsolicited requests for off-label information with a focused response that is objective, balanced, accurate and substantiated and supported by scientific evidence.

### Joint Meetings with Sales Representatives

Joint visits with commercial colleagues are for the purposes of introductions. Commercial discussions and scientific exchange need to occur outside of joint interactions. The best practice would be to divide the meeting. This means the MSL and the SR should be visiting the HCP with different agendas and objectives.

Key Principles

- An external stakeholder can request off-label information either directly from the MSL or through the medical information process
- Such requests need to be documented in an appropriate way

## Summary

The role of the MSL is pivotal to the communication, collaboration and exchange of scientific information with both internal and external stakeholders. MSLs represent the scientific face and force of the pharma industry, connecting companies with the medical community that include a range of stakeholders. Through the exchange of highly credible, unbiased, scientific and clinical information, MSLs can build and foster important scientific credibility with these external experts thus bringing relevant, timely and actionable insights to the company. They can also support clinicians by discussing the latest scientific breakthroughs. In order to build credibility with healthcare professionals, MSLs need to maintain independence from sales and promotional-based activities.

As illustrated in current times of social distancing and the disrupted access to face-to-face interactions with peers, digital channels have become critical tools to achieve and maintain engagement and relationships. This technological shift may mark a more permanent approach to how MSLs perform their role in the future, although it remains to be seen how much of the traditional ‘meeting-in-person’ will be replaced with virtual meetings, which is likely to vary between different companies. Regardless, the need for MSLs to be technologically savvy will become increasingly important to allow for versatility and to accommodate the preference of the experts following their own experience of communicating through digital platforms. The best practices outlined in this paper are embedded in principles that are applicable to both.

## Abbreviations

APPA: Australian Pharmaceutical Medical and Scientific Professionals Association
IFAPP: International Federation of Associations of Pharmaceutical Physicians and Pharmaceutical Medicine
MAPS: Medical Affairs Professional Society
MSLS: Medical Science Liaison Society
